# Effect of methomyl on hepatic mixed function oxidases in rats

**DOI:** 10.4103/0253-7613.43162

**Published:** 2008-08

**Authors:** Jyotsna A. Patil, Arun J. Patil, Ajit V. Sontakke, Sanjay P. Govindwar

**Affiliations:** Department of Biochemistry, BLDEAS Shri B.M. Patil Medical College, Bijapur, India; 1 Krishna Institute of Medical Sciences, Deemed University, Karad, India; 2 Shivaji University, Kolhapur, India

**Keywords:** Aminopyrine N-demethylase, cytochrome P_450_, methomyl, mixed function oxidase, toxicity

## Abstract

**Objective:**

To study the effect of the methomyl on mixed function oxidase system in rats.

**Materials and Methods:**

The effect of the methomyl on mixed function oxidase was studied using different dosages, durations and sex. Microsomes were isolated using the calcium precipitation method. The levels of cytochrome P_450_ , and cytochrome b_5_  were determined using extinction coefficient of 91 and 85 mM^-1^  respectively. The activities of drug metabolizing enzymes, hemoglobin content, liver function enzymes, and serum cholinesterase activity were assayed by using standard methods.

**Results:**

Intraperitoneal administration of methomyl (4 mg/kg body weight) showed significant decrease in the level of cytochrome P_450_ , and the activities of aminopyrine N-demethylase and aniline hydroxylase on the third day of the treatment. Methomyl (4 mg/kg) treatment of old male rat and adult female rat also showed a decrease in the level of cytochrome P_450_ , and aminopyrine N-demethylase activity. The serum samples from methomyl treated rats (male and female), when analyzed for alanine aminotransferase (SGPT) and aspartate aminotransferase (SGOT) as markers of the liver toxicity, showed significant increase in the activity. The activities of SGPT and SGOT were significantly higher in the treated rats (2 and 4 mg/kg) than in the control group. A significant decrease in the level of hemoglobin and serum cholinesterase activity was observed, when there was an increase in the dose level. A significant increase was observed in alkaline phosphatase activity at all dose levels.

**Conclusion:**

Methomyl influences mixed function oxidase and creates abnormality of liver functions in the rats. This effect depends on the dose and duration of methomyl.

## Introduction

Lannate is widely used for the control of a large variety of insects (leafhoppers and thrips), on a wide range of crops viz. fruits, vines, hops, vegetables, grains, soybeans, cotton, and ornamentals throughout the world.[1] The active ingredient in Lannate is a methomyl, (S-methyl-N-(methyl carbomyl)-thioacetimidate), a compound of the oxime carbamate group. Methomyl has been classified as a pesticide of category-1 toxicity. The maximum concentration allowed for foliar application of methomyl to grapes is 0.9 lb per acre.[2] It is an insecticide of low chronic, but high acute toxicity, which acts by direct contact or following ingestion through the stomach.[3]

Metabolic pathway for methomyl in the rat includes the displacement of the S-methyl moiety by glutathione and enzymatic transformation to produce a mercapturic acid derivative. Another pathway involves hydrolysis to give S-methyl-N-hydroxy thioacetimidate, which is rapidly broken down to carbon dioxide.[1] Methomyl is rapidly metabolized to CO_2_  and acetonitrile. Methomyl treated rats showed histopathologic changes in kidney, and spleen of male and female rats. Similarly, enzymatic alterations of acetyl cholinesterase and liver glucose-6 phosphate dehydrogenase were also observed.[3]

The mixed function oxidase system plays an important role in the metabolism of many compounds, including fatty acids, steroids, alkanes, polycyclic carcinogens, drugs and environmental pollutants. Methomyl is a potent inhibitor of acetyl cholinesterase, in both insects and mammals. The signs of toxicity are those expected from a cholinesterase inhibitor, like profuse salivation, lacrimation, tremors, abnormal posture, pupil constriction, diarrhea and prostration.[1] The aim of the present study is to assess the effect of methomyl on mixed function oxidase system, blood parameters which include liver enzymes and hemoglobin.

## Materials and Methods

### Animals

Three-month-old male (weighing 200-230 g, 80-100 days old) and female (weighing 200-230 g, 80-100 days old) Wistar rats were housed in standard cages and were given standard laboratory diet (Hindustan Lever Ltd., Mumbai) and tap water *ad libitum*. The animals were used after clearance from the Institutional Animal Ethics Committee.

### Chemicals

Nicotinamide adenine dinucleotide phosphate (reduced form) NADPH, glucose 6 phosphate, Glucose-6 phosphate dehydrogenase, aminopyrine, aniline hydrochloride and HEPES were obtained from Sigma Chemical Co. (St Louis, USA). Methomyl (40%) (Lannate) was obtained from Du Point, USA. Potassium chloride, ethylenediamine tetra acetic acid, sodium deoxycholate, trichloroacetic acid, sucrose, calcium chloride and other chemicals were of analytical grade and obtained from Qualigen Fine Chemicals (Mumbai, India). Standard kits of alanine aminotransferase, aspartate aminotransferase and alkaline phosphatase were obtained from Span diagnostics, India. Cholinesterase kit was obtained from Labcare, India. 

### Animal treatment

Adult male rats (weighing 200-230 g) were divided into four groups of six animals each. Group 1 received an equivalent amount of saline and served as control. Animals from group 2, 3, and 4 were given methomyl 1, 2, and 4 mg/kg body weight i.p. respectively, between 8: 00 a.m. and 9:00 a.m., for three consecutive days. In the second set of experiments, adult male rats were divided into four groups. Animals from group 2, 3, and 4 were injected with 4 mg/kg body weight methomyl for one, three, and five successive days. The volume injected into the rats of body weight 200 g was 1 ml. In another experiment, adult female rats and old male rats (weighing 350-450 g, 8 months old) were divided into two groups of six animals each. Group 2 of each category received methomyl (4 mg/kg body weight, i.p., for three successive days), and group 1 of each category received an equivalent amount of saline and served as the control group.

### Preparation of hepatic microsomes

The rats were sacrificed by cervical dislocation, 24 hours after the last dose. Their livers were perfused in situ with ice cold 1.15% KCl containing 0.05 mM EDTA, rapidly excised, blotted dry, weighed, minced and homogenized with two volumes of ice cold 0.25 M sucrose solution, in a Potter-Elvehjem type homogenizer. The homogenate was centrifuged at 10,000 × g for 10 min, in a refrigerated centrifuge (REMI C-24). The microsomes were isolated by the procedure described by of Cinti *et al.*[4] The microsomal pellet was suspended in phosphate buffer (0.1 M, pH 7.4) and the suspension was used for the microsomal enzyme assays. Microsomal protein content was measured by the Biuret method, using bovine serum albumin as the standard.[5]

### Microsomal enzyme assay

The microsomal electron transport components (cytochrome P_450_ and cytochrome b_5_) were determined using Hitachi UV-Visible double beam recording spectrophotometer, by the procedure of Omura and Sato.[6,7] Cytochrome c reductase activity was determined using the method recommended by Masters *et al.*[8] Aminopyrine N-demethylase activity was assayed according to the procedure described by Schenkman *et al.*[9] Formaldehyde liberated during N-demethylation was estimated by the procedure described by Nash.[10] Aniline hydroxylase assay was performed using the procedure reported by Govindwar and Dalvi.[11]

### Liver function enzymes

Alanine aminotransferase and aspartate aminotransferase activity was measured using the Reitman and Frankel[12] method. Alkaline phosphatase activity was measured using Span diagnostic kits.[13] Cholinesterase activity was measured using the kinetic test method of Knedel *et al.*[14] 

### Hemoglobin content

Hemoglobin was measured using HLS-hemosafe reagent. The color obtained was measured colorimetrically at 530 nm. 

### Analysis of data

Statistical analysis was done by using one-way analysis of variance and Tucky Krammer post test. The level of significance was set at 0.05.

## Results

Administration of methomyl at different dose levels (1, 2 and 4 mg/kg) resulted in a significant decrease in hepatic microsomal protein content (28, 31 and 19%, respectively). A decrease in cytochrome P_450_  content (34, 34 and 19%) was observed at all three dosages (1, 2 and 4 mg/kg). A significant decrease in the activity of aminopyrine N-demethylase was observed by 50 and 40% at 2 and 4 mg/kg dose, respectively. Cytochrome c reductase activity was increased by 14% at 4 mg/kg dose level. The activity of aniline hydroxylase was decreased by 27% at 4 mg/kg dose level. No significant change was observed in cytochrome b_5_  at all the three doses [Table 1]. The selected dose 4 mg/kg was used to study the inhibitory effect of methomyl for different durations. A significant decrease was observed in the level of cytochrome P_450_  (37, 42 and 37%), activities of aminopyrine N-demethylase (31, 51 and 31%), and aniline hydroxylase (47, 71 and 17%), at all durations (one, three and five days, respectively). No significant change was observed in microsomal protein, cytochrome b_5_  and the activity of cytochrome c reductase [Table 2].

**Table 1:** Effect of different doses of methomyl (i.p., for three days) on microsomal protein, electron transport components and drug metabolizing enzymes of the adult male rats (weighing 200-220 g)

*Parameters*	*Methomyl treatment i.p., for 3 days, mg/kg*				*One -way ANOVA*
		
	*Control*	*1*	*2*	*4*	
Microsomal protein	18.73	13.53	12.83	15.10	*P*<0.0001
(mg/g liver)	±0.36	±0.73***	±0.36***	±0.47***	F=27.4
Cytochrome b_5_	0.19	0.16	0.20	0.19	*P*<0.0287
(nmole/mg protein)	±0.002	±0.005	±0.014	±0.003	F=5.13
Cytochrome P_450_	0.41	0.27	0.27	0.33	*P*<0.001
(nmole/mg protein)	±0.001	±0.005***	±0.003***	±0.001***	F=488.9
Cytochrome c reductase^a^	32.27	35.73	32.80	36.93	*P*<0.0035
	±1.27	±0.21	±0.97	±0.80**	F=6.3
Aminopyrine N-demethylase^b^	3.8	3.2	1.9	2.3	*P*<0.001
	±0.18	±0.21	±0.04***	±0.35***	F=14.8
Aniline hydroxylase^c^	1.10	1.35	1.17	0.80	*P*<0.024
	±0.08	±0.10*	±0.17	±0.09*	F=3.9

a nmole of cytochrome c reduced/min/mg microsomal protein

b nmole of formaldehyde liberated/min/mg microsomal protein

c nmole of p-aminophenol formed/min/mg microsomalprotein, Values are mean of three experiments ± SEM, six rats in each group. Those marked with asterisks differ significantly from the control value

* *P*< 0.05

** *P*< 0.01

*** *P*< 0.001 (by ANOVA and Tucky Krammer post test)

**Table 2:** Effect of duration of methomyl (4 mg/kg i.p., for up to five days) on microsomal protein, electron transport components and drug metabolizing enzymes of the adult rats

*Parameters*	*Days of the treatment*				*One -way ANOVA*
		
	*0*	*1*	*3*	*5*	
Microsomal protein (mg/g liver)	13.50	15.77	16.00	12.20	*P*<0.046
	±1.1	±1.4	±0.9	±0.5	F=3.18
Cytochrome b_5_	0.27	0.25	0.29	0.28	*P*=0.3472
(nmole/mg protein)	±0.01	±0.02	±0.01	±0.02	F=1.17
Cytochrome P_450_	0.48	0.30	0.28	0.30	*P*<0.0001
(nmole/mg protein)	±0.018	±0.002***	±0.006***	±0.009***	F=79.1
Cytochrome c reductase^a^	25.1	31.3	26.7	24.2	*P*=0.197
	±1.4	±2.8	±2.5	±2.7	F=1.70
Aminopyrine N-demethylase^b^	4.50	3.11	2.20	3.09	*P*<0.0001
	±0.22	±0.18**	±0.11***	±0.31***	F=19.10
Aniline hydroxylase^c^	1.13	0.60	0.33	0.93	*P*<0.0001
	±0.05	±0.01***	±0.04***	±0.02***	F=108.9

a nmole of cytochrome c reduced/min/mg microsomal protein

b nmole of formaldehyde liberated/min/mg microsomal protein.

c nmole of p-aminophenol formed/min/mg/microsomal protein, Values are mean of three experiments ±SEM, six rats in each group.

Those marked with asterisks differ significantly from the control value *P<0.05

** *P*<0.01

*** *P*<0.001 (by ANOVA and Tucky Krammer post test)

Further, treatment of methomyl (4 mg/kg) of the old male rat showed a significant decrease in cytochrome P_450_  (43%), cytochrome b_5_ (51%), cytochrome c reductase (7%), aminopyrine N-demethylase (50%), and aniline hydroxylase (27%). Methomyl (4 mg/kg) treatment of adult female rat also showed a significant decrease in the level of cytochrome b_5_  (21%), and the activities of cytochrome c reductase (42%), aminopyrine N-demethylase (26%), aniline hydroxylase (43%), as compared to the control group. [Table 3].

**Table 3:** Effect of methomyl (4 mg/kg body weight, i.p., three days) on microsomal protein, electron transport component and drug metabolizing enzymes of old male rats and adult female rats

*Parameters*	*Old male*		*Adult female*		*One way ANOVA*
			
	*Control*	*Test*	*Control*	*Test*	
Microsomal protein	19.55	18.80	11.76	10.90	*P*<0.0001
(mg/g liver)	±0.9	±1.6	±0.8	±0.6	F=18.97
Cytochrome b_5_	0.41	0.20	0.19	0.15	*P*<0.0001
(nmole/mg protein)	±0.04	±0.01***	±0.01	±0.01*	F=28.82
Cytochrome P_450_	0.35	0.20	0.24	0.21	*P*<0.0001
(nmole/mg protein)	±0.002	±0.007***	±0.017	±0.014	F=35.19
Cytochrome c reductase^a^	35.80	33.30	40.17	23.07	*P*<0.0003
	±0.8	±0.5*	±1.7	±4.1**	F=10.22
Aminopyrine N-demethylase^b^	4.50	2.26	4.50	3.30	*P*<0.0001
	±0.20	±0.11***	±0.20	±0.29*	F=26.47
Aniline hydroxylase^c^	1.23	1.80	0.80	0.45	*P*<0.001
	±0.07	±0.06***	±0.09	±0.01***	F=18.10

a nmole of cytochrome c reduced/min/mg microsomal protein

b nmole of formaldehyde liberated/min/mg microsomal protein

c nmole of p-aminophenol formed/min/mg/microsomal protein, Values are mean of three experiments ± SEM with six rats in each group. Those marked with asterisks differ significantly from the control value

* *P*< 0.05

** *P*< 0.01

*** *P*< 0.001 (by ANOVA and Two-tail post test)

The effect of methomyl at different doses on liver function enzymes, acetyl cholinesterase and hemoglobin content was studied. Increased activity of aspartate aminotransferase by 9 and 26% (at 2 mg/kg), and 8 and 39% (at 4 mg/kg) was observed in male and female rats, respectively. Increased activity of alanine aminotransferase by 12 and 14% (at 2 mg/kg), and 20 and 24% (at 4 mg/kg) was observed in male and female rats, respectively. Total protein content was decreased significantly in male (by 13, 22 and 30%), and in female rats (by 14, 11 and 23%), at all the three doses. Serum alkaline phosphatase activity was increased significantly in male (14, 29 and 39%) and female (33, 61 and 87%), at all the three doses. Different doses depressed acetyl cholinesterase activity significantly by 15, 28 and 35% in male and 46, 52 and 66% in female, respectively. Hemoglobin content showed decreased levels by 16, 25 and 29% in male rat and 21, 21 and 29% in female rat at all the three doses [Tables 4 and 5]. 

**Table 4:** Effect of different doses of methomyl (i.p., for three days) on liver function enzymes, serum cholinesterase and hemoglobin content on male rats

*Parameters*	*Groups (methomyl treatment mg/kg body weight)*				*One-way ANOVA*
		
	*Control*	*1*	*2*	*4*	
SGOT	34.7	35.7	37.9	40.9	*P*<0.0001
(u/l)	±0.55	±0.30	±0.41**	±0.93***	F=21.17
SGPT	44.6	47.3	50.0	53.6	*P*<0.0001
(u/l)	±0.85	±0.92	±0.73**	±1.40***	F=14.55
Serum total protein	2.30	2.02	1.80	1.60	*P*<0.0001
(g/dl)	±0.04	±0.08**	±0.04***	±0.04***	F=32.33
Alkaline phosphatase	20.5	23.4	26.4	28.6	*P*<0.0001
(KA units)	±0.39	±0.60**	±0.59***	±0.19***	F=55.67
Serum cholinesterase	1311	1120	949	848	*P*<0.0001
(u/l)	±48	±31**	±27***	±31***	F=33.32
Hemoglobin	11.40	9.60	8.60	8.10	*P*<0.0001
(g /dl)	±0.15	±0.28***	±0.07***	±0.13***	F=69.19

Values are mean of three experiments ± SEM with six rats in each group. Those marked with asterisks differ significantly from the control value *P<0.05

** P<0.01

*** P<0.001 (by ANOVA and Tucky Krammer post test)

**Table 5:** Effect of different doses of methomyl (i.p., for 3 days) on liver function enzymes, s erum cholinesterase and hemoglobin content on female rats

*Parameters*	*Groups (methomyl treatment mg/kg body weight)*				*One-way ANOVA*
		
	*Control*	*1*	*2*	*4*	
SGOT	36.8	43.5	46.6	51.0	*P*<0.0001
(u/l)	±1.04	±2.10*	±1.80***	±0.26***	F=16.206
SGPT	40.3	43.0	46.0	50.0	*P*<0.0001
(u/l)	±0.55	±0.63	±1.40**	±0.84***	F=20.591
Serum total protein	2.20	1.90	1.96	1.70	*P*<0.0190
(g/dl)	±0.005	±0.03	±0.19	±0.06*	F=4.175
Alkaline phosphatase	14.3	19.0	23.0	26.7	*P*<0.0001
(KA units)	±0.42	±0.37**	±0.37***	±1.50***	F=42.036
Serum cholinesterase	2470	1343	1193	844	*P*<0.0001
(u/l)	±24	±35***	±37***	±102***	F=145.82
Hemoglobin	12.70	10.06	10.00	9.06	*P*<0.0001
(g /dl)	±0.54	±0.46**	±0.42**	±0.27***	F=13.022

Values are mean of three experiments ± SEM with six rats in each group. Those marked with asterisks differ significantly from the control value

* P<0.05

** P<0.01

*** P<0.001 (by ANOVA and Tucky Krammer post test)

## Discussion

The objective of this study is to examine the effect of carbamate (methomyl) on the liver microsomal drug metabolizing enzyme system, liver function enzymes and on hemoglobin content. Male Wistar rats receiving a dose of methomyl (4 mg/kg) developed signs of toxicity within 2 min. The maximum severity viz. muscle fasciculation and convulsions developed within 5-10 min and lasted for about 30 min. The rats were free from obvious toxicity within 90 min.[15] A decrease in the level of cytochrome P_450_  and the activity of drug-metabolizing enzymes at higher dose level [Table 2] and in old male rats [Table 3], indicates the destruction of cytochrome P_450_. This may have also been due to the inhibition of heme synthesis. Many pesticides including organochlorine and organophosphorous compounds have been reported to inhibit the activity and alteration in the expression of various cytochrome P_450_  isoforms e.g. parathion inactivates the CYP3A4 isoform during its oxidative biotransformation. These changes may increase the sensitivity of cells against reactive endogenous metabolites or other xenobiotics. Thus it has been postulated that the inhibition of cytochrome activity by organophosphorous compounds contribute to the development of Parkinson's disease by rendering the neurons more sensitive to toxic metabolites of neurotransmitters.[16]  Methomyl also inhibited the activity of mixed function oxidases in quails. The biochemical mechanism responsible for this inhibition is uncertain. These results are important for the identification of the mechanisms of acute toxicity of these compounds, especially in view of the lack of bibliographic information on the effects of this insecticide on the MFO system.[17]  An increase in the activity of alanine aminotransferase, and aspartate aminotransferase activity showed the dose-dependent phenomenon. Altered liver enzyme activities have been reported among pesticide workers exposed to organophosphorous pesticide alone or in combination with organochlorine or other pesticide.[18]  Serum glutamate oxaloacetate transaminase and serum glutamate of pyruvate transaminase activity are known toxicity markers in the study of hepatotoxicity of chemicals.[11]  An increase in the activity of these enzymes is termed as the early recognition of toxic hepatitis. A significant increase in these enzyme activities was observed at higher dose, indicating liver damage. However, reports show that the enzyme activity returned to normal values by the end of the intoxication period, suggesting repair of the damaged liver cells. An earlier report shows increased enzyme activity on the 17^th^  day due to lower oral dose and from the 4^th^  to 17^th^  day due to higher dose. The elevation pattern was almost restored to normal thereafter, which may be attributed to the inhibition of the circulating enzyme by methomyl and/or its metabolites.[3]

Methomyl is known as a potent cholinesterase inhibitor and the animals exhibited tremors, salivation, and irregular breathing a few minutes after being dosed. Serum cholinesterase activity was significantly inhibited at higher dose. Generally, the recovery of the enzyme activity is attributed firstly to the reversible interaction between the insecticide and acetyl cholinesterase, and secondly to rapid metabolism, and elimination of the compound.[3]  Methomyl aerosol showed marked suppression on plasma ChE but no cumulative effect on red cell ChE in male rats by repeated inhalation.[3]  Organophosphorous (e.g. parathion and malathion) and N-methyl carbamate (e.g. carbaryl, aldicarb) insecticides are believed to act by inhibiting AChE activity. They do so by acting as pseudosubstrates and forming a covalent adduct with active site serine. This results in the accumulation of acetylcholine in the synapse, overstimulation of AChE receptors and, death by respiratory failure. Parathion and malathion become much more potent AChE inhibitors, following oxidation in a reaction catalysed by cytochrome P_450_  monooxygenases.[16] 

Hemoglobin content significantly decreases in all the three doses. Decreased hemoglobin content, hematocrit value, erythrocyte, and leucocyte count was reported in old male rats on the administration of both methomyl and trichlorofon insecticides.[19]

In conclusion, it may be said that a decrease in cytochrome P_450_  content and the activity of drug metabolizing enzymes due to methomyl treatment indicate the inhibition of mixed function oxidases in dose and duration dependent manner. A significant increase in SGOT, SGPT, serum proteins and alkaline phosphatase indicates liver damage. Methomyl inhibits cholinesterase activity and causes toxicity. A decrease in hemoglobin may be because of the inhibition of heme synthesis.
